# Popular Fallacies

**Published:** 1870-02

**Authors:** 


### POPULAR FALLACIES.

There is fun sometimes in running full tilt against popular opinion; that is, fun to the person who knows he is right, and fun to the populace when they know the man is wrong. There are some things so plain that it is really difficult to go about proving them. There are assertions so palpably absurd that when made the individual making them is looked upon with pitying or indignant contempt. We hope the reader won't faint at the following announcements, nor get so mad as to order the discontinuance of the *Journal*, nor that the editor will be considered a fit subject for a lunatic asylum.

We sometimes feel assured that we know something to be true, arising from the fact of our ignorance of an existing acknowledged fact, which demonstrates the groundlessness of the assurance. The man who grew up on a small island, had never left it, and had never seen beyond any thing but a waste of waters, knew, as he thought, that there was no other land in the world, but was convinced to the contrary when a ship came and took him to another country.

How many persons knew, how many scientific men demon. strated to themselves that messages could never be sent across the Atlantic by telegraphic wires.

How many knew that it was a fatal blunder on the part of the manufacturers of the first Atlantic cable to have made two parts twist in different directions at the point of union, and yet that same wire is working as well as the other to this day.

Again; there are some opinions which we have cherished all our lives, seem so self-evidently true, that we have never felt it necessary to look around for a reason, and if asked to do so, it may put us to some little trouble to find one that is conclusive. He is the grandest man who is willing to investigate any proposition with calmness and to be led wherever truth directs.

With these forefenders, we say:—

1. Early rising is not healthful.
2. The habitual use of tea and coffee at every regular meal is healthful.
3. Tight lacing does not tend to produce consumption, but to cure it.
4. Sugar and candy do not injure the teeth or health.
5. Gymnasiums are not beneficial.
6. Life Insurance is not a public good.
7. Cold water is worse than wine at meals.
8. Sweet milk is not a healthful food.
9. A bad cold never can cause (originate) consumption.

The American people, as a class, are too lazy to think; pure plodding deliberation, a patient and careful examination of any subject, is by common consent voted as an insufferable bore. We had a great deal rather that some one outside of ourselves should do our thinking for us, and give reasons made to hand. It is a common trait in us to look at a desirable object at a distance and jump across the intervening space, rather than deliberately pick our steps one by one through the mire that lies between us and the coveted goal. Hence the innumerable failures in business, failures of life! And to make a failure of life, how fearful! We are too prone to feel that it is not worth while to disturb public opinion. But it can not only do no good to cover up a lie, but it must be productive of unmixed evil if it be a practical thing, and will do evil to the end of time, if not corrected.

Suppose, for example, that we all believe that sweets cause the teeth to decay, then we pay too much attention to their avoidance, and too little to guard against greater injuries to the teeth, want of cleanliness. It is proposed to consider these "Popular Fallacies" from time to time. In this February number the habitual use of tea and coffee at each meal as healthful is considered.

